# Six solutions for clinical study data sharing in Germany

**DOI:** 10.1186/s12874-025-02560-y

**Published:** 2025-05-24

**Authors:** Evgeny Bobrov, Christina Habermehl, Daniel Strech, Tracey Weissgerber, René Bernard

**Affiliations:** 1 QUEST Center for Responsible Research, Berlin Institute of Health at Charité– Universitätsmedizin Berlin, Berlin, Germany; 2 Cluster of Excellence NeuroCure, Charité– Universitätsmedizin Berlin, corporate member of Freie Universität Berlin, Humboldt Universität zu Berlin, Berlin, Germany; 3 Present Address: Leibniz Institute of Freshwater Ecology and Inland Fisheries (IGB), Berlin, Germany; 4 Present Address: CNC-UC, Center for Neuroscience and Cell Biology, Center for Innovative Biomedicine and Biotechnology, University of Coimbra, Coimbra, Portugal

**Keywords:** Clinical trials, Clinical studies, Clinical research, Data sharing, Data reuse, Secondary data use, Data protection

## Abstract

Sharing clinical study data is endorsed by many funders and journals, international policy frameworks, and patients. Reuse of clinical study data demonstrably improves health research, and emerging technologies may enhance the value derived from shared data. Unfortunately, clinical research has failed to harness the transformative power of data sharing, and sharing remains the exception. This opinion piece focuses on the massive obstacles to sharing clinical study data in Germany, which results in very low sharing rates, wasted resources, and frustration among local researchers and international partners. We argue that this sharing crisis demands immediate and concerted action. As a remedy, we propose six feasible steps to boost clinical study data availability in Germany, derived from our experience consulting researchers and exploring solutions with international partners. Our recommendations target ethics committees, trial registries, infrastructures, and governance, while addressing data protection concerns. These measures must be flanked by further actions to foster data sharing skills and knowledge as well as, most importantly, the provision of appropriate incentives. Nevertheless, the proposed changes would be a breakthrough for clinical study data sharing in Germany, removing barriers regarding infrastructures, awareness, legal uncertainty, and responsibilities.

## Teaser

Sharing clinical study data is widely endorsed and improves health research, but nevertheless remains the exception. We address obstacles to study data sharing in Germany and propose six solutions. These target ethics committees, trial registries, infrastructures, and governance. Taken together, they would boost study data sharing in Germany.

## Background

Funders, institutions, and policy makers strongly encourage researchers to share their data, allowing others to validate results and explore new questions. Data reuse avoids unnecessary duplication, permits meta-analyses, and honors patients’ contributions, including the burdens and risks of study participation. Funders and journals are increasingly making data sharing mandatory [1–3]. Patients and healthy volunteers want their data to be shared and utilized, to benefit their patient group and society at large [4, 5]. Shared clinical study data is frequently reused and demonstrably improves health research through trial reanalysis [6, 7], meta-analysis [8], and methods development [9]. The value of reusable data is increasing with the advent of artificial intelligence and data spaces, such as the European Health Data Space. However, thus far, only a small subset of existing clinical data has been shared, and thus clinical research fails to harness the transformative power of shared data. A comprehensive meta-analysis has shown that only 2% of research articles in medical and health science share underlying data [10]. Here, we argue that this sharing crisis demands immediate action by various stakeholders. As remedy we propose six feasible actions to boost the availability of clinical study data in Germany.

Our experiences are based on consulting in over 50 cases with regard to diverse aspects of data sharing, including consent and de-identification, data quality and usability, and sharing venues and processes. In these cases, where researchers at one of the largest University Medical Centers in Europe wanted to share clinical study data, confirm that potential data providers in Germany face many obstacles. Thus, it is unsurprising that data sharing rates are very low. Despite widely observed high motivation from researchers to share valuable data, very few datasets were ultimately shared. The few datasets that were shared were subjected to protracted, time-consuming agreements to achieve legal compliance. Data minimization procedures introduced during this process substantially reduced the potential for reuse, while legal liability still resided with data creators. As we experienced repeatedly in our consulting, it was not just researchers at our own institution who abandoned their data sharing ambitions due to frustrations with the complex red tape and the discrepancy between political expectations and on-the-ground impossibilities. Germany’s stringent data protection laws have made sharing medical research data challenging, leading to frustrations among international collaborators, including German researchers abroad. These complexities could be a contributing factor in Germany’s declining participation in global clinical research [11]. The German Medical Research act (*Medizinforschungsgesetz* [12]), which came into effect on October 2024, is in part designed to address this issue.

The lack of legal harmonization and its downstream consequences are the primary obstacle to clinical data sharing in the EU [13], and this is all the more true for Germany. While the General Data Protection Regulation (GDPR) offers a useful legal framework to protect the personal data of individuals, the current interpretation and handling of GDPR in Germany creates high bureaucratic burdens and, even more detrimental, legal uncertainty. This complexity and uncertainty associated with data sharing is further compounded by clinical investigators potentially bearing sole responsibility [14] for any future legal consequences resulting from their decision to share data. Ultimately, this prevents clinical data sharing for the good of patients as an unintended consequence. In contrast, discussions with Austrian and Dutch stakeholders, who also operate under the GPDR, demonstrated that institutional and national infrastructures can facilitate clinical data sharing. Such proven best practices need to be transferred to the German clinical research landscape to avoid being left behind.

## Definition, scope, and limitations

The present piece focuses on the critical need to share clinical study data. Insofar as clinical trials are included, we only consider investigator-initiated trials (IITs), while excluding pharma-sponsored trials. We do not address primary care data, for which the German Medical Informatics Initiative (MII) is currently establishing processes for data standardization and access. While we focus on clinical studies, some solutions may apply more broadly to research with human participants or human-derived material. Importantly, this piece is not about sharing fully anonymized data, as true anonymization is typically unattainable as long as the study center retains a link to the individual. In this regard, anonymization itself is also considered by some experts a form of data processing that requires consent, although this might have been solved by the Health Data Use Act (*Gesundheitsdatennutzungsgesetz*,* GDNG*). There is an ongoing discussion about the most fitting legal grounds for study data processing [15, 16]. The positions of different EU countries and the EDPB with regard to requiring consent are inconsistent [17, 18]. However, in Germany consent for study data is widely accepted, recommended, and in most cases mandatory [19–21]. A German research data law (*Forschungsdatengesetz*) addressing this question might be forthcoming [22], and the recent adoption of the EHDS regulation [23] might shift the balance towards other legal grounds. However, while EU regulations are binding for member states, it remains to be seen if and when this regulation will alter both the conceptual approach to consent and the concrete assessments of data sharing requests in Germany. For the above reasons, we view consent as the only regularly feasible path to sharing clinical study data in Germany at this time.

Thus, we focus here on the problems and possible solutions for the consent-based sharing of clinical study data from publicly funded research in Germany.

Most of the authors (RB, EB, CH) are data managers, and this opinion is a practice-led appeal for a discussion on how to bring needed improvements for clinical study data sharing to the German research landscape. Many different types of expertise, however, are needed to fully define and implement the proposed actions. This opinion piece does not claim to do justice to valuable viewpoints of data protection officers, medical informatics experts, ethics committees, patient advocates, and other stakeholders. Furthermore, the solutions proposed here need to be supported by further measures, including dedicated research and training on trial data sharing [24, 25]. Cultural change is also needed to make data sharing the norm, as many researchers are reluctant to share data and do not perceive data sharing as best practice [26, 27]. However, the best way to change the current culture is to take concrete steps that address current obstacles and empower those who want to share data. We acknowledge that a lack of incentives to share data [28] is a major obstacle to such a change, which must be addressed.

## Problems and solutions

### Problem: intent to share data is not part of applications to research ethics committees (RECs)

There is an ethical obligation to share data, as codified in the International Ethical Guidelines for Health-related Research Involving Humans, Guideline 24 [29] of the Council for International Organizations of Medical Sciences (CIOMS) and the World Health Organization (WHO). The International Committee of Medical Journal Editors (ICMJE) similarly sees this as an ethical obligation [30]. Despite these statements, as well as an often-expressed desire of patients to have their research results reused with appropriate safeguards [4, 31, 32], data sharing is not included in REC applications [33].

**Solution: Include data sharing in REC applications**: Data sharing is both an ethical and scientific necessity and should therefore be integrated into the risk-benefit assessment carried out by RECs. While data protection rights create challenges, it is crucial to set the right balance between participant rights and broader societal benefits. Legal data protection issues fall outside the scope of RECs, but these can still address ethical aspects of handling personal data, which includes assessing the applicant information with regard to data sharing plans. Independently of the REC application, each study must also submit a data protection concept or data protection impact assessment, where legal concerns can be handled. Including data sharing in REC applications does not conflict with legal obligations but reframes the issue, promotes data sharing, and prompts investigators to plan for data sharing from the start. Simply asking about data sharing, even without formal requirements, signals that it should be the norm and, as a result, raises its profile. RECs should align their assessments regarding data sharing to ensure consistency, such that no additional barriers are created [34].

### Problem: consent statements typically do not include data sharing by default

Currently, data sharing beyond the study center and defined partners is not typically a part of informed consent documents. As consent is the standard legal pathway for data sharing in Germany, researchers who do not integrate data sharing into their consent forms are typically unable to share data when the wish or need arises later.

**Solution: Mainstream consent to share data**: To provide a path for subsequent reuse, data sharing for future research must become a standard part of consent forms. The main challenge is drafting consent that is broad enough to allow sharing with unknown future partners or for new research purposes. Given that data will be shared either in de-identified form or pseudonymized under contractual agreements, this approach is both legally and ethically sound. The MII’s broad consent model already uses this for sharing primary health care data [35]. When participants are patients, consent should be obtained using the MII’s study module for contract-based sharing [36]. Appropriate consent statements must be included in cases where broad consent is not used, participants are not patients of University Medical Centers (UMCs), or de-identified data will be shared openly. These statements should become standard practice and be included by default. They can be complemented by meta-consent processes which are currently being developed, in which patients consent to individual instances of data use [37, 38].

### Problem: central questions of data protection are not answered

A central point of contention we encountered is the handling of study participants’ rights under GDPR. For instance, the extent to which rights (e.g. right to erasure, right to rectification) can be waived by consent remains unclear. This is central for both data sharing approaches:

- Case 1: A principal investigator (PI) wants to openly share a de-identified dataset version, which, however, still contains personal data according to GDPR. Study subjects have consented to this. At the same time, under GDPR, subjects have certain rights, e.g. to request data erasure. For openIy shared data, erasure is technically impeded, and it also runs counter to scientific integrity and poses organizational challenges. A similar logic applies to other rights under GDPR, e.g. right to rectification. In such cases, can study subjects waive certain rights under GDPR with respect to open sharing of data, which would not affect the data in the study center, which are still linked to direct identifiers?
- Case 2: A PI wants to share pseudonymized data through an agreement which includes a mandatory deletion clause, i.e., the data user will delete the data after a defined period. Then, while data copies exist at partner institutions, the study subject makes use of his rights under GDPR, e.g. by requesting erasure. In such an arrangement, can study subjects consent that the sole obligation of study PIs with respect to subjects’ rights concerning data copies held by data users is to inform the users about the request? In other words, can the study PI avoid being responsible for ensuring the actual data deletion?

**Solution: Clarification of central consent-related questions**: All stakeholders in the clinical study data sharing landscape in Germany need legal clarity on the data provider’s obligations when data sharing agreements include a clause mandating that the reuser delete data at a specified time point after the dataset has been shared (e.g., the date when a study participant requests that his or her data be deleted). Stakeholders need to know whether data providers are legally responsible for ensuring that reusers comply with such clauses. Releasing data providers from the responsibility of ensuring reuser compliance would reduce practical barriers to data sharing. Similarly, it is essential to clarify whether rights under GDPR can be waived if patients consent to openly sharing versions of datasets which are de-identified to reach a minimal risk of re-identification. Researchers and study subjects need legal certainty in their rights management. Standard clauses for potential waivers would be extremely valuable. Statistical and technical alternatives to consent-based data sharing, like synthetic data [39, 40] (also known as data swapping), code-to-data [41], and secure multi-party computation [42] are being explored in the German medical research context [43]. While these methods are effective in certain use cases, they are not a universal replacement for sharing original data. Therefore, solutions to the problems described above are urgently needed.

### Problem: responsibility for data sharing resides with researchers only

Despite mandatory consultations with data protection offices and, at times, additional experts, the ultimate responsibility and legal liability for data sharing rests with researchers. In a setting where the institution does not explicitly assume this responsibility, researchers are understandably hesitant to take these risks, fearing severe consequences or, as some describe it, “having one foot in prison”. While these concerns might be overstated, the perceived risks and consequences are a major obstacle to data sharing. If institutions view data sharing as part of their mission, as many claim, they must demonstrate this by shifting legal responsibility from individual researchers to the institution itself. The legal literature seems to make no distinction between institutions and individual employees with regard to responsibilities under GDPR [44, 45], presumably locating such questions at the level of internal relationships. While this might be technically correct, as long as responsibility under conditions of uncertainty is not spelled out, this does not solve the problem from the researcher perspective. Indeed, a multitude of institutional research data policies which explicitly address researcher responsibilities suggest otherwise, e.g [46, 47].

**Solution: Create processes for institutional decision-making on data sharing**: As part of the MII’s mission to make primary health care data available across all German UMCs, Data Use and Access Committees (DUACs) have been introduced [48]. These committees already have established processes for data access based on nationally agreed-upon frameworks. However, the MII DUAC arrangement still requires a “principal investigator” who carries the responsibility of releasing data. For study data, we propose to create “data clearing offices”, as implemented successfully in Vienna Medical University [49]. This office offers a well-defined process for making data sharing decisions. Such a construct could be introduced and adapted in Germany. The MII-DUACs’ remit can be extended to take on this role, and shift responsibility for data sharing decisions to the institution. Importantly, these ‘study DUACs’ should handle both data sharing requests coming from outside the institution and those coming from inside, where institutional researchers seek to share data. It is also crucial to develop blueprints for the necessary processes at the national level through the MII and/or NFDI4Health, and to ensure that DUACs operate transparently.

### Problem: central data storage and access solutions are lacking

In addition to legal considerations, effective data sharing requires long-term, quality-assured, and standardized data storage and access. Although decentral data storage does not inherently inhibit data sharing, centralized data infrastructures can substantially enhance data availability. First, while study PIs should remain central to the data-sharing decision, long-term access is easier if data is in a location designed to be accessible to a trusted group beyond the PI. Second, centralized infrastructures support data standardization, ranging from metadata collection to format choice unification, which improve data interoperability and reusability.

**Solution: Provide access through data integration centers**: The MII has established Data Integration Centers (DIZ) at all German UMCs as an infrastructure for primary health data sharing. These DIZs are already open to clinical trial data, provided that patients have consented to data sharing [48]. Since January 2023, the coordination of the DIZs has transitioned to the Network of University Medicine (NUM) that works in close collaboration with the MII. Given their presence at all UMCs and the already established processes, DIZs are ideally positioned to serve as the default infrastructure for storing clinical study data. However, their capacity to manage data collected without the broad consent study module remains uncertain. Expanding the scope of DIZs to broadly include clinical study data would require additional expertise and resources. Beyond addressing technical and organizational challenges, DIZs should fully embrace this role and actively promote it within their institutions. Successful models for study data sharing, such as the Dutch Data Archiving and Networked Services (DANS) [50] can provide valuable insight.

### Problem: trial registries lack information on data availability

Clinical trial registries generally provide limited information on data sharing, often framing it in ways that do not encourage considering whether to provide data access: The German Clinical Trial Registry (DRKS) entry form, for example, asks if participant data will be made “anonymous”. This is a misleading question given the technical challenges and legal inconsistencies concerning full anonymity. Questions about making data “public” are similarly unclear. This discourages trial PIs from committing to data sharing. The German Central Health Study Hub (HSH) [51] also lacks consistent data sharing information as it aggregates from other sources. While institutions have begun to monitor preregistrations for clinical trials, not all studies are preregistered [52], and data sharing practices are rarely tracked. Registries do not follow up with users, and therefore, updates to the data availability status are infrequent and shared data cannot be located, which exacerbates the lack of sharing-related information.

**Solution: Reframe data sharing information in registries**: We specifically urge the DRKS to revise its data sharing-related questions to better align with data sharing procedures available to researchers. Registries and meta-registries like the HSH should not only indicate whether data is available but also detail the specific application process for data access. In addition, registries should point study PIs to data sharing through automatic reminders. The recent EHDS Regulation could in the long run constitute a legal requirement for a more comprehensive collection and exposure of study metadata. Data availability information should be considered right from the start when implementing the EHDS Regulation.

## Conclusion

Unlocking the potential benefits of clinical study data sharing will require contributions from all stakeholders in the German health research system. While data sharing and reuse are currently seen as complicated, optional, and rare, they must become feasible, expected, and common. Many changes are required to remove fundamental obstacles and make data sharing the norm. In our view, enabling data sharing and facilitating reuse is essential to benefit patients and to value their investment in and commitment to medical research. While data protection is needed to protect patients and maintain trust, we argue that it is important to shift the emphasis from risk elimination to risk minimization. This will allow us to accelerate progress while upholding data protection principles. Inaction with regard to data sharing also introduces risk. If standard guidelines and procedures for data sharing are absent, any data sharing that occurs runs the risk of being of poor quality and may pose risks to the data subjects. In summary, the sharing of clinical research data in Germany is a complex, but tractable problem. We hope that the solutions proposed here might stimulate discussion and action to remove barriers and normalize data sharing to the benefit of patients and the German medical research community.

## Data Availability

No datasets were generated or analysed during the current study.

## Abbreviations

DIZ: *Datenintegrationszentrum* (Data Integration Center)
DRKS: *Deutsches Register Klinischer Studien* (German Clinical Trial Registry)
DUAC: Data use and Access Committee
EDPB: European Data Protection Board
EHDS: European Health Data Space
GDPR: General Data Protection Regulation
GDNG: *Gesundheitsdatennutzungsgesetz* (Health Data Use Act)
HSH: Health Study Hub
MII: *Medizinformatik-Initiative* (German Medical Informatics Initiative)
NFDI4Health: *Nationale Forschungsdaten-Infrastruktur* (National Research Data Infrastructure, NFDI) for Personal Health Data
REC: Research Ethics Committee
UMC: University Medical Center
